# Supplementary material for: Are carbon emissions trading and green financial instruments synergistic? -Comprehensive quantitative research based on content analysis
Source: PLoS One. 2024 Mar 7;19(3):e0298601. doi: 10.1371/journal.pone.0298601 (PMC10919601; doi:10.1371/journal.pone.0298601)
Supplement: S1 Data — (ZIP) [file pone.0298601.s001.zip › Data/地级市Z_F工作报告.zip!/╔╜╬≈/╠1⁄2╘¡╩╨/└┤╫╘╣1⁄2╓┌║┼í╛┬φ┐╦╩2╛▌═°í┐_2017.docx]

各位代表：

现在，我代表市人民政府，向大会报告工作，请予审议，并请政协委员和列席人员提出意见。

一、过去五年工作回顾

全市上下在省委、省政府和市委的正确领导下，认真贯彻落实习近平总书记系列重要讲话精神和治国理政新理念新思想新战略，按照省委“一个指引、两手硬”重大思路和要求，迎难而上，砥砺前行，积极适应经济发展新常态，坚持稳中求进总基调，统筹做好稳增长、促改革、调结构、惠民生、防风险各项工作，努力实现“两个走在前列”，较好完成市十三届人大一次会议确定的经济社会发展主要目标任务。

——经济运行持续稳定增长。经济发展首位度有所提升，占全省的22.9%。2016年实现地区生产总值 2955.60亿元，年均增长7.8%；规模以上工业增加值571.81亿元，年均增长7.2%；固定资产投资 2027.71亿元，年均增长14.6%；一般公共预算收入282.69亿元，年均增长10.1%；社会消费品零售总额 1666.24亿元，年均增长11.4%；外贸进出口总额133.14亿美元，年均增长9.4%；城镇居民人均可支配收入29632元，年均增长9.1%；农村居民人均可支配收入14591元，年均增长10.2%。

——产业结构优化升级初见成效。特色农业稳步发展，亿元以上农业龙头企业17家。非煤产业占规模以上工业增加值的90.2%，富士康手机制造、太重高铁轮对、阳煤化机制造等一批项目建成投产，新能源、新材料、节能环保、电子信息、食品药品等新兴接替产业占比由34.2%提高到70.9%。万达综合体、绿地中央广场、茂业天地等一批服务业项目加快推进，服务业增加值占地区生产总值的62.6%。

——发展新动能逐步积累。推进科技创新，全社会研究与试验发展经费年均增长6.3%。新增5个国家级科技企业孵化器和7个国家级重点实验室、工程技术中心；国家级高新技术企业533家，高新技术产业增加值占地区生产总值的8.4%。创建国家小微企业创业创新基地城市示范，推进大众创业、万众创新，各类市场主体35.3万户，建成清控等8个国家级众创空间，2016年实现民营经济增加值 1701.22亿元,年均增长9.8%。鼓励发展多层次资本市场，新三板挂牌企业37家，占全省三分之二，金融业增加值占地区生产总值的14.8%。

——城市基础设施建设成效明显。新建改建府东府西街、五一路、并州路、南内环街、长风街、学府街、东峰路等城市主次干道160余条，改造支路近400条，建设总里程750余公里；建成中环路、卧虎山路、建设路、阳兴大道、滨河西路、南沙河路、马练营路等快速路213公里，快速交通体系初步形成。新建北中环桥、摄乐桥两座汾河大桥，完善提升柴村桥、双塔街、学府街与滨河东路立体互通体系。太原高铁南站建成投入使用，地铁2号线全面开工建设。敷设供水、供热、燃气、电力、通讯等各类地下管线8453公里，公共供水普及率达到85.15%，燃气普及率达到98.65%。推进80多个城中村和108片棚户区总计10万余户改造，人民群众居住条件进一步改善。

——省城环境质量继续改善。集中供热扩网1.3亿平方米，覆盖率达到90%，替代分散燃煤采暖锅炉3198台，拆除城中村烟囱43700余个，关停搬迁太化、煤气化、狮头水泥等污染企业301家，年减少燃煤近千万吨。古交兴能、太二、太钢等电厂366万千瓦热电燃煤机组完成超低排放改造。老旧建筑节能改造近千万平方米。淘汰老旧机动车和黄标车27.3万辆，2500辆公交车全部使用天然气，8292辆出租车整体更新为纯电动汽车；投放公共自行车4.1万辆，日租车人数超过50万人次。新建城南污水处理厂和晋阳污水处理厂。饮用水源地水质达标率保持100%。二级以上天数由162天增加到232天。加强东西北山生态建设，完成营造林168万亩，森林覆盖率达23%；新建晋阳公园、和平公园等13个公园和46个小游园，道路绿化375万平方米，建成区绿化覆盖率、绿地率、人均公园绿地面积分别达到41.7%、36.7%、11.9平方米。

——民生保障能力不断提高。累计民生支出1467亿元，占一般公共预算支出的82.7%。每年城镇新增就业保持在10万人左右，城镇登记失业率控制在4%以内。城乡居民养老、医疗保险和低收入群体基本生活保障实现制度全覆盖，城乡低保5年7次提标，分别提高49%和84%，“新农合”实现应保尽保。扶贫攻坚首战首胜，7万农村贫困人口实现脱贫。推进教育医疗资源标准化、均衡化、优质化发展，新建改扩建五中、成成、一外、二外等学校606所，小学、初中90%以上学区实现就近入学，普通高中实现网上录取；新建改扩建市中心医院、妇幼医院、人民医院等医疗机构47所，可新增床位5000余张。建成城乡老年日间照料中心448个，开工建设儿童福利院。持续举办太原国际马拉松赛，积极承办全国第二届青运会。全国双拥模范城创建实现“八连冠”。人防工作获得全国先进城市称号。推进“平安省城”建设，安全生产和社会稳定形势总体平稳向好。

——文化事业文化产业加快发展。推进晋祠景区完善提升，太山龙泉寺、明太原县城、青龙古镇保护性开发取得阶段性成果，文殊寺、普光寺、圆通寺、关帝庙等府城历史文化遗存保护加快实施。改扩建太原图书馆，建成开放太原美术馆、赵梅生美术馆，成功举办第十二届全国美术作品展览雕塑作品展、2015太原国际雕塑双年展，连续四年在太化工业遗址举办国际青年金属雕塑创作营活动。晋剧《傅山进京》《于成龙》和舞剧《千手观音》等作品入选国家艺术基金项目。建成西山文化创意、清徐陈醋等特色文化产业园。

——政府自身建设不断加强。严格执行政府决策议事规则和工作程序，自觉接受人大依法监督、政协民主监督，认真听取各民主党派、工商联、无党派人士建言献策，累计办理人大代表建议1341件、政协提案2800件，办复率100%。积极推行权力清单和责任清单制度，推进“放管服”改革，市级行政权力事项精简54%。认真落实政府党组全面从严治党主体责任，严格执行八项规定，坚决反对“四风”，加大监察审计力度，加强党风廉政建设。扎实开展党的群众路线教育实践活动、“三严三实”专题教育和“两学一做”学习教育，干部队伍作风进一步转变，干事创业氛围更加浓厚。

过去五年，全市经济社会发展取得的成绩，是在省委、省政府和市委坚强领导下，市人大、市政协监督支持下，全市广大干部群众攻坚克难、团结奋斗的结果。在此，我代表市人民政府，向全市人民、驻并部队、武警官兵和公安干警，向各位代表、各位委员和社会各界人士，表示崇高的敬意和衷心的感谢！

我们也清醒认识到，当前太原正处在滚石上山、爬坡过坎的关键时期，还存在不少困难和问题。主要表现在：发展水平不高，发展速度不快，首位度不够，特别是工业不大不强，经济下行压力仍然较大；创新驱动、转型升级任务艰巨，传统产业改造提升不快，引领发展的战略性新兴产业发展不足；城市基础设施欠账较多，城市管理水平不高，影响人民群众的幸福感；省城环境质量改善形势严峻，燃煤污染依然突出，全面治理亟需加强；发展环境不优、服务意识不强、行政效能不高，少数干部不作为、慢作为、庸懒怠和腐败现象依然存在。我们要直面问题，担当负责，努力以改革发展的新举措认真解决。

二、今后五年工作目标任务

今后五年，是全面深化改革、全面实现小康社会目标的决胜期，是实施创新驱动、推动转型升级的攻坚期，也是实施“十三五”规划、实现太原振兴崛起的重要战略机遇期。我国经济发展进入新常态，供给侧结构性改革深入推进，“一带一路”“京津冀协同发展”等重大战略深入实施，省委、省政府建设山西转型综改示范区重大战略加快落实。我市正站在振兴崛起的历史关头，处于转型升级的历史拐点，面临干事创业的历史机遇，我们要坚定信心、攻坚克难、开拓进取，努力实现省、市第十一次党代会提出的目标任务。

未来五年经济社会发展的主要目标是：经济结构达到新优化。创新驱动、转型升级成效显著，战略性新兴产业加快发展，先进制造业、信息产业、新能源、新材料、现代服务业成为主引擎。地区生产总值年均增长7.5%以上，第一产业增加值年均增长3%以上，规模以上工业增加值年均增长7.5%以上，服务业增加值年均增长8%以上、占地区生产总值60%以上。省会城市的首位度进一步提升，地区生产总值占全省的30%左右。民生改善体现新水平。城乡居民人均收入与经济增长同步，城乡收入差距进一步缩小。城镇登记失业率控制在4%以内，力争2018年实现贫困人口脱贫、贫困县摘帽。2020年全面完成170个城中村拆迁改造任务。教育、文化、社保、医疗、住房等公共服务体系更加健全，基本公共服务均等化水平得到提高。安全生产形势持续稳定好转，人民群众的幸福指数明显提升。

城市功能完成新提升。城市空间布局更加优化，基础设施不断完善，主次干道、支路级次配比科学合理，立体化综合交通体系基本形成。地铁1、2、3号线基本建成，初步形成地铁网络体系。城市综合管廊和海绵城市建设成效明显，城市防洪排涝体系强大健全，雨污分流体系完整，城市综合承载能力和城市管理水平全面提高。道路更加通畅，环境更加优美，城市更加宜居，在全国省会城市中位次前移，凝聚力、竞争力和影响力进一步增强。

文化建设出现新进步。社会主义核心价值观更加深入人心，人民群众思想道德素养、科学文化素质、健康生活水平明显提高，进入全国文明城市行列。公共文化基础设施更加健全，现代公共文化服务体系逐步建立。文化软实力明显提升，文化旅游产业发展壮大，战略性支柱产业的框架形成，旅游目的地的目标基本实现，国家历史文化名城影响力进一步扩大。文化产业增加值年均增长20%，占地区生产总值的7%左右。

生态环境得到新改善。生态绿色屏障基本形成，生产方式、生活方式绿色低碳水平显著提高。市区污水处理、集中供热、垃圾无害化处理实现100%全覆盖。PM2.5浓度逐年下降，市区优良天气率达到80%左右。森林覆盖率达到30%以上，建成区绿化覆盖率达到42%左右，天蓝、山绿、水清的目标基本实现，城乡人居环境全面改善。

改革开放实现新突破。转型综改示范区建设取得重大成果，重点领域和关键环节改革取得突破性进展，发展动力和活力显著增强，要素集聚、体制改革、创新活跃、生产力解放，对外开放的深度广度进一步拓展。太原成为开放高地、投资热土、增长引擎，在全省对外开放格局中的带动作用进一步增强。

法治政府建设取得新进展。人民民主不断扩大，法治建设更加完善，合法权益得到切实保障，司法公平正义进一步彰显，法治政府、法治社会一体化推进。办事依法、遇事找法、解决问题用法、化解矛盾靠法的法治意识提升到一个新高度。政府治理体系和治理能力不断提高，社会各项事务治理更加规范化、程序化、制度化。

三、2017年工作安排

今年政府工作的总体要求是：深入贯彻习近平总书记系列重要讲话精神和治国理政新理念新思想新战略，统筹推进“五位一体”总体布局，协调推进“四个全面”战略布局，认真落实省、市第十一次党代会总体部署和省委、市委十一届二次全会暨经济工作会议精神，按照省委“一个指引、两手硬”重大思路和要求，坚持新发展理念，坚持稳中求进工作总基调，坚持深化供给侧结构性改革与深化转型综改试验区建设有机结合，坚持以提高发展质量和效益为中心，以转型综改示范区为引擎，全面实施创新驱动、转型升级战略，全力做好稳增长、促改革、调结构、惠民生、防风险各项工作，努力实现“两个走在前列”，以优异成绩迎接党的十九大胜利召开。

主要预期指标是：地区生产总值增长8%左右，规模以上工业增加值增长7.5%左右，固定资产投资增长7%左右，社会消费品零售总额增长7.5%左右，一般公共预算收入增长3.5%左右，城乡居民人均可支配收入分别增长7.5%和8%左右，居民消费价格涨幅控制在3%左右，城镇登记失业率控制在4%以内，圆满完成省下达的约束性指标任务。

（一）以供给侧结构性改革为主线，推进创新驱动、转型升级

用改革的办法深入推进“三去一降一补”。扎实有效淘汰落后产能，严格执行环保、能耗、质量、安全等相关法律法规和标准，坚决淘汰技术落后、污染严重、安全隐患较大的煤焦冶电企业。有效处置“僵尸企业”，落实职工安置政策。分类施策去库存，通过改变商业模式、降低运营成本等多元化途径消化商业写字楼库存。进一步采取减税降费、降低“五险一金”缴费比例、全面清理规范政府性基金、推进大用户直供电等举措，促进企业降成本。

用供给侧结构性改革的力量振兴实体经济。实体经济是发展的根基。要以创新驱动为主动力、转型升级为主引擎、实体经济为主战场，大力发展电子信息、先进装备制造、新材料、新能源、节能环保等战略性新兴产业，努力形成若干千亿、百亿级产业集群，构建供给侧结构性产业新优势。着力抓好富士康手机智能化制造和维保、江铃重汽整车及发动机、比亚迪新能源汽车、太重地铁盾构和风电装备、中车地铁装备、太钢 T800 碳纤维和轮轴钢、大明不锈钢深加工、晋西春雷高精度铜合金、中电科二所碳化硅半导体等重点项目，以项目促转型，以创新添活力，建立高质量、多层次、宽领域的有效供给体系。弘扬工匠精神，厚植工匠文化，创建质量强市，推动经济发展进入质量时代。深入实施《中国制造2025》，推进大数据、云计算、物联网运用，推动传统产业生产、管理和营销模式变革。实施煤焦冶电等传统产业技术改造、智能改造、绿色改造。发挥军工基地产业优势，创新军民产业融合机制，开展军民协同创新，推动军民融合深度发展。

创新是第一动力，人才是第一资源。功以才成，业由才广。认真落实省委《关于深化人才发展体制机制改革的实施意见》，推进人才强市战略，构建科学、开放、高效的人才管理体制。以识才的慧眼、爱才的诚意、容才的雅量、用才的胆识，创新以增加知识价值分配为导向的人才政策，促进创新要素向高端人才流动、高端人才向转型综改集聚。加强与科研机构、高等院校产学研用深度结合，引进站在行业科技前沿、具有国际视野和能力的领军人才，加快新材料、人工智能、集成电路、生物制药、第五代移动通信等技术研发成果转化，培育高科技引领的产业集群。

着力提升服务业发展水平。开展“互联网+”产业集群行动，加快建设国家小微企业创业创新基地城市示范，打造面向大众的“双创”全程服务体系，推进大众创业、万众创新，发展新制造、新技术、新业态。大力发展现代服务业，推动生产性服务业向价值链高端延伸、生活性服务业向精细化高品质延伸。积极发展总部经济、数字经济、互联网服务等新兴产业，培育壮大金融、科技服务等重点产业，推进电子商务示范工程，发展跨境电子商务。着力抓好华润万象城、苏宁广场、润恒农产品冷链物流、万科现代仓储、传化公路港等重大服务业项目建设。完成农村信用社体制改革，设立支撑创新驱动、转型升级两个百亿元产业基金。开工建设12万平方米大型会展中心，创新太原低碳发展论坛等重点会展办会机制，培育会展品牌，打造全省会展核心区。推进农业供给侧结构性改革。发展具有省城特色的城郊型农业，重点抓好设施农业、观光农业、休闲农业、龙头企业、中药材基地、农产品加工销售产业链，开发和发展功能食品，打造农村一二三产业融合发展新格局，加快培育农业农村发展新动能。

（二）以关键环节和重点领域改革为突破口，增强内生发展动力和优势

把改革放在更加突出的重要位置，勇挑最重的担子，啃最硬的骨头，扑下身子，狠抓落实。加快推进基础性、关键性改革，以改革先人一招实现发展快人一步。

持续推进政府职能转变。深化简政放权、放管结合、优化服务改革。全面实行清单管理制度，以清单管理推动简政放权。实施政府部门权力和责任清单，清单之内依法规范履职，清单之外市场主体选择。对政府自身进行“正面清单约束”，对市场进行“负面清单管理”，减少政府的自由裁量权，增加市场的自主选择权。深化商事制度改革，实行多证合一、证照分离。完善事中事后监管制度，实现“双随机、一公开”监管全覆盖，推进综合行政执法。发展“互联网+政务服务”，实现政府信息系统互联互通，公共资源交易、土地招拍挂网上办理。创造公平竞争、开放创新的制度环境，实现审批最少、流程最优、体制最顺、机制最活、效率最高、服务最好，让企业和群众实实在在感受到“放管服”改革成效，增强人民群众改革获得感。

深化投融资体制改革。坚持问题导向，与中央要求“对表”、与发达地区对标、与国际惯例对接，大幅减少投资项目前置审批，大幅缩减政府核准投资项目范围。对核准类项目，只保留选址意见、土地征用以及重特大项目环评审批；对备案类项目，不得设置任何前置条件，实行属地办理。创新企业投资管理，探索建立多评合一、多审合一、多图联审、联合验收等新模式，除烦苛之弊、施高效之策、开便利之门。创新拓宽投融资渠道和方式，大力推广利用 PPP、 债券、基金等市场化融资模式，降低政府负债风险。推进多层次资本市场发展，培育上市企业，积极发展主板、创业板、新三板、区域性股权交易市场。

继续推进财税体制改革。落实和完善营改增政策，进一步减轻企业税费负担。深化财政预算改革，推进政府预决算公开，超前谋划和科学编制预算，合理安排财力，及时拨付到位，盘活沉淀资金，提高资金使用效率。公款姓公，每一笔钱都要花在明处、用出实效。

深化国企国资改革。以提高核心竞争力和资源配置效率为目标，形成有效制衡的公司法人治理结构、灵活高效的市场化经营机制。推动国资监管体制向管资本为主转变，确保资产保值增值、法人主体放开经营。彻底剥离企业办社会职能，解决历史遗留问题，瘦身健体、提质增效、健康发展。

积极争取创建国家可持续发展议程创新示范区。对标世界先进水平，加强国际交流合作，以可持续发展理念为引领，发挥科技创新的核心作用，破解资源型城市路径依赖的难题，打造可复制、可推广、可持续发展的现实样板。

更好激发非公有制经济活力。鼓励企业家创新创业，保护企业家合法权益，尊重企业家社会地位，按“亲”“清”二字处理好政商关系，努力形成亲商、安商、容商、富商的良好氛围。进一步优化政务服务环境，公正公平对非公有制人士综合评价，宽容失败，激励创新，促进发展。持续深化“万名干部入企服务”活动，真心帮助，真情服务，真正解决企业发展的瓶颈障碍，做大做强民营经济支撑的半壁江山。

（三）以转型综改示范区建设为载体，打造转型升级新引擎转型综改是习近平总书记为山西发展指明的金光大道。省委、省政府建设转型综改示范区的重大决策部署，为我市转型综改、创新驱动创造了历史性的重大机遇，我们要解放思想，抢抓机遇，举全市之力管理好、支持好、服务好示范区建设。

加快启动起步区建设。按照“整合改制扩区调规”要求，用好用足国家调整土地利用规划、核减基本农田的政策，加快小店、清徐、阳曲土地利用总体规划调整和永久基本农田划定，落实起步区土地规划指标，尽快完成3.5万亩土地征收任务，确保4月份启动起步区建设。

优化产业布局。高点起步、高端发展、高标准规划建设管理，打造聚集先进生产要素的政策洼地和发展高地。加大招商引资引智引技力度，力争在智能制造、电子信息、新能源、新材料、节能环保、生物医药等领域，引进一批高质量的大项目、好项目，推进高端化、智能化、绿色化、品牌化战略性新兴产业发展。巩固、提升、拓展示范区已经形成的产业基础，实现存量扩能提质、增量倍增发展，持续发力保增长。推动区区融合，深化不锈钢园区产业规划，优化空间布局，围绕不锈钢深加工形成产业集群，做大做强产业链。

创新体制机制。借鉴国家自贸区可复制、可推广的改革试点经验，营造法治化、国际化、便利化营商环境，创造改革创新发展新优势。建立专业化、市场化、国际化管理运行机制，实行领导班子任期制、全员岗位聘任制和绩效工资制。加强全面深化改革开放各项措施系统集成，加快具有较大影响力的科技创新步伐，大胆试、大胆闯、自主改，当好改革开放排头兵、创新驱动先行者、率先发展领头雁。

（四）以建设宜居宜业幸福城市为目标，推进城市基础设施建设和城市管理水平提升

统筹推进太原都市区现代化建设，坚持建管并重，注重增强城市功能，彰显城市特色，着力打造创新、开放、品质、智慧、绿色、低碳、平安、幸福城市，提升省会城市核心竞争力和知名度、美誉度。

发挥规划先行引领作用。规划是百年尺度，城市以规划论输赢。要有全球化视野、特色化定位、现代化品质，彰显太原显山露水、绿色宜居的自然格局，文化厚重、人文荟萃的历史底蕴，功能完备、活力迸发的都市风范。顺应城市发展规律，以新的城市文化发展理念，提升规划的前瞻性、科学性和综合性，年内完成“五规合一”试点工作。高起点规划晋阳湖片区，加强汾河两岸及主要干道的规划控制和城市设计，点线面结合、山水城一体，打造太原历史文化底蕴和现代都市风貌的靓丽名片。

继续加大城市基础设施建设力度。树立大交通、大格局意识，优化提升市域道路综合体系。开工建设太原西北二环高速，拉大城市框架。完成环城高速与北中环东延、南中环西延等互通工程，推进太长高速太原段改线，实现滨河东路南延发展。发展轨道交通，加快地铁2号线建设，启动3号线、1号线建设。新建改造东中环北延、西中环南路、学府街东延等50条300公里城市快速路和主次干道，45条60公里支路。继续推进公交都市建设。加大城市综合管廊、海绵城市建设力度。推进220千伏汾东等24项供电工程，提升完善电力输配网络体系。加快太原南站东广场、汽车客运东南站建设及功能配套。高标准完成高铁沿线环境综合整治。继续优化提升“铁、公、机”，更加重视“岸、港、网”建设。改造提升南坪头、马庄、七府坟等水库、缓洪池以及太榆退水渠防洪设施，以百年一遇标准夯实防洪排涝体系。

大力推进城中村和棚户区改造。新启动30个城中村和25个集中连片棚户区改造，新建开工棚户区保障房2.96万套，基本建成2.2万套。推进县城、集镇和城边村改造提升，建设特色小镇和美丽乡村。加快城中村、棚户区改造拆迁清零，尽快完成安置回迁，切实兑现承诺，提高政府的公信力。安置回迁是良心工程，各级政府都不能透支人民群众的信任，要让被安置群众尽快告别租住日子，在住有所居中创造新生活。

深入开展城市管理全面提升行动。推进交通秩序、环境卫生、户外广告、打违拆违等十项综合整治，全面改善市容市貌。2017年全面攻坚，2018年巩固提升，形成常态化管理和长效工作机制。推进智慧城市建设，运用大数据、互联网等科技手段，提升城市现代化、精细化、智能化管理水平。

扎实做好全国第二届青运会筹办工作。这是我市历史上第一次承办全国性综合体育盛会，是提升我市城市基础设施建设和管理水平、塑造城市文明形象的重大契机。全市上下要树立主人翁精神，精心做好二青会比赛场馆和青运村建设，积极开展全民体育健身运动，大力加强宣传教育，携手提升文明素养，全面推进“五城联创”，以文明、开放、热忱、美丽的城市形象，迎接二青会胜利召开。

（五）以生态文明发展理念为引领，持续加大环境污染治理力度

实行最严格的生态环境保护制度，坚守生态保护红线，开展铁腕治污行动，压实各级党政环保责任，强化环保问责和执法，坚决打好蓝天保卫战，确保省城环境质量持续改善。

全面改善空气质量。以“控煤、治污、管车、降尘”为重点，坚持问题导向，采取坚决措施，确保 PM2.5 浓度均值下降9.09%， SO2 指标下降10%，力争在全国74个重点监控城市排位稳定向好。全力解决燃煤污染，加快实施集中供热全覆盖，新增供热能力3500万平方米，实现市区151个35吨以下分散采暖燃煤锅炉全部替代。按照企业为主、政府推动、居民可承受的方针，全力推进86个城中村、188个城边村、37个集中连片棚户区分散燃煤整治，通过采取整村整片拆除改造一批、集中供热一批、电化一批、气化一批、新农村建设改造一批等措施，完成10万户清洁供暖，全年减少燃煤100万吨。继续推进既有建筑节能改造。严控工业污染排放，提升绿色企业标准，对达不到排放标准的，停产限期达标改造；太原一电厂和清徐、阳曲等不达标焦化厂停产，太钢原料场和渣场全封闭改造；加强太钢和二电厂周边综合整治，实现原煤、原材料铁路运输。涉煤企业要逐步退出建成区，夯实绿水青山的基础。全面控制扬尘污染，严格落实绿色施工标准，实施在线全程实时监控管理，对不达标工地重处重罚，停工整治；渣土清运全程清洁化，湿法拆迁、密闭运输、车辆清洗、达标运营。彻底淘汰黄标车，取缔非法经营黑车，施工车辆全面达标准入，提高燃油品质，严控机动车尾气排放。加强露天烧烤、秸秆焚烧等面源污染整治，建成区餐饮业全部加装高效油烟净化装置。推进畜禽粪便治理。加强对大气污染源解析和雾霾形成机理研究，提高治理的科学性和精准性，积极应对气候变化，强化预警和应急措施。

加强水生态建设。综合整治北涧河、北沙河、北排洪渠、玉门河、虎峪河、九院沙河、冶峪河、风峪河等8条河流，源头治理、蓄水调洪，雨污分流、河水复清，快速交通、绿色长廊，连片改造、全面提升。推进晋阳湖和晋阳湖湿地为中心的大生态建设，打造山水一体、河湖连通、古今交融、人文自然辉映的一流生态功能区，彰显“三面环山、一水中分、一湖点睛、九河环绕”的水韵龙城格局。全面推行河长制，健全生态保护补偿机制。实行最严格水资源管理制度，强化饮用水源地环境治理与保护，确保汾河水库水质稳定达标。加快汾东污水处理厂、垃圾焚烧电厂、餐厨污泥处理等重点工程建设，加强中水回用。积极推进建筑垃圾资源化利用，加强土壤污染防治与修复。

加强绿化造林和生态建设。继续加大东西北山绿化力度，推进荒山绿化，完成营造林43万亩。加快迎泽公园、动物园、植物园、摄乐公园等公园新建改造，启动西山国家矿山公园、北部国家农业公园建设，新建30个街头小游园，建成区绿化覆盖率、绿地率、人均公园绿地面积新增0.5%、0.5%和0.3平方米。

（六）以提升城市文化软实力为核心，大力发展文化事业文化产业

文化是城市的灵魂，是一个城市凝聚力、创造力、影响力的重要源泉。要坚持社会主义先进文化前进方向，树立高度的文化自觉和文化自信，为全市经济社会发展凝聚强大的精神文化力量。

大力发展文化旅游产业。完善提升晋祠景区环境综合整治，加快晋阳古城大遗址保护和明太原县城保护性开发，力争青龙古镇和太山龙泉寺景区10月1日全面开放。启动双塔历史文化大景区建设、狄仁杰文化主题公园、太化工业文明遗存展示、钟楼街和东三道巷历史街区保护开发，打造城市文化名片和文化客厅。继续推进府城历史文化片区保护，重点保护好文庙和崇善寺、天主教堂和拱极门、文瀛湖和五一路、南华门和教场巷等四大历史文化片区，留住城市记忆，强化实物例证支撑。大力完善旅游设施和服务，发展乡村、休闲、全域旅游。

积极发展文化事业。坚持用中国梦和社会主义核心价值观凝聚共识、汇聚力量，推动文艺繁荣发展，努力创作集思想性、艺术性、观赏性于一体的精品佳作。弘扬社会主义时代精神，建设诚信社会，尊崇诚实守信。推动基本公共文化服务标准化、均等化发展。加快推进太原博物馆陈列布展，市图书新馆5月1日对外开放。积极开展“书香太原”全民阅读系列活动，不断满足人民群众精神文化需求。

着力培育文化市场主体。积极引进文化旅游龙头企业，打造精品景区和旅游路线。促进文化产业与互联网融合发展，发展创意设计、数字传媒、动漫游戏等文化创意产业，创新体制机制，培育发展新兴文化业态。建设华夏历史文明主题公园，打造文化领军企业和知名文化品牌，提升文化旅游业整体发展水平。

（七）以保障和改善民生为重点，统筹推进各项社会事业坚持以人民为中心的发展思想，顺应人民群众对美好生活的向往，把保障和改善民生作为一切工作的根本出发点和落脚点，夯实基础，兜牢底线，切实解决人民群众普遍关心的突出问题，把发展硬道理更多体现在增进人民福祉上。

坚决打好脱贫攻坚战。推动特色产业发展、易地移民搬迁、劳务输出、金融、教育和健康扶贫，动员社会力量参与扶贫，强化龙头企业引领，实施贫困村整体提升工程，建立健全稳定脱贫长效机制，落实脱贫攻坚责任制，增强脱贫群众内生动力。严格贫困退出机制和评估考核机制，确保脱贫得到群众认可，经得起历史检验。再战再胜，今年完成2万人脱贫任务。

继续推进教育医疗资源标准化、均衡化、优质化发展。建设公办幼儿园20所，完成五中、成成、育英等6所中学新校和实践基地建设，加快推进职教园区建设。全面推行公立医院改革，基本完成市中心医院、市人民医院、市妇幼医院和省妇幼医院等6所新院建设。加强基层医疗服务保障能力建设，统筹三医联动，推进县乡医院一体化管理，逐步建立分级诊疗制度，提升人民群众健康水平。全面推进养老服务业发展，建设高标准示范性老年福利院，逐步建立以居家养老为主体、以社区养老为依托、以机构养老为补充的社会养老服务创新体系。加快推进康宁医院、救助站和儿童福利院建设，完善社会救助服务体系。鼓励创业带动就业，促进城乡居民收入增长。实施全民参保登记计划。加快采煤沉陷区搬迁建设和治理修复等民生工程。加强国防和后备力量建设，提高新形势下双拥工作水平。

全力维护社会安全稳定。严格落实党政同责、部门监管、企业主体安全生产责任，加强重点行业领域安全监管，坚决杜绝重特大安全生产事故发生。积极创建国家食品安全城市，切实加强对食品药品的监督管理。推进平安省城建设，加强和创新社会治安综合治理，依法打击违法犯罪行为，营造平安和谐的社会环境。

（八）以为人民服务为宗旨，全面加强政府自身建设牢固树立“四个意识”，坚决维护以习近平同志为核心的党中央权威，自觉在思想上政治上行动上同党中央保持高度一致，旗帜鲜明讲政治、脚踏实地干实事、全心全意为人民。

深入贯彻全面依法治国要求，严格遵守宪法，尊崇法治、敬畏法律、依法行政，坚持科学、民主、依法决策。自觉接受人大及其常委会依法监督和人民政协民主监督，主动接受社会和舆论监督，认真听取人大代表、政协委员、民主党派、工商联、无党派人士和各人民团体的意见，政府所有工作都要充分体现人民意愿、维护人民利益、接受人民监督。依法管理宗教事务，提高宗教工作水平，更好组织和凝聚宗教界人士、信教群众，形成太原振兴崛起的合力。

深入推进党风廉政建设和反腐败工作，落实“一岗双责”，担负起政府党组管党治党主体责任。带头执行《准则》《条例》，把好用权“方向盘”，系好廉洁“安全带”，激浊扬清，扶正祛邪，忠诚、干净、担当，自觉为营造良好政治生态履职尽责，推动全面从严治党向纵深发展。

推进“两学一做”学习教育常态化制度化，加强政治文化建设，坚定共产主义理想信念。严格落实“13710”工作制度，以上率下、马上就办，提高工作效率和服务质量。重大问题和事项盯住不放，一抓到底，限时办理。落实市委激励担当和合理容错两个《办法》，为担当的干部担当，对负责的干部负责，给干事者鼓劲、创业者撑腰，弘扬真抓实干、埋头苦干、以实绩论英雄的时代精神，以实干推动发展，以实干赢得未来。

各位代表，新征程催人奋进，新使命艰巨光荣。让我们更加紧密地团结在以习近平同志为核心的党中央周围，在省委、省政府和市委的坚强领导下，撸起袖子加油干，扑下身子抓落实，扬鞭奋起创新业，以优异成绩迎接党的十九大胜利召开！
